# Multiple Team Membership, Performance, and Confidence in Estimation Tasks

**DOI:** 10.3389/fpsyg.2021.658827

**Published:** 2021-05-31

**Authors:** Oana C. Fodor, Petru L. Curşeu, Nicoleta Meslec

**Affiliations:** ^1^Department of Psychology, Babeş-Bolyai University, Cluj-Napoca, Romania; ^2^Department of Organization, Open University of the Netherlands, Heerlen, Netherlands; ^3^Department of Organization Studies, Tilburg University, Tilburg, Netherlands

**Keywords:** multiple team membership, estimation tasks, group-to-individual transfer of learning, groups, learning, individual performance

## Abstract

Multiple team membership (MTM) is a form of work organization extensively used nowadays to flexibly deploy human resources across multiple simultaneous projects. Individual members bring in their cognitive resources in these multiple teams and at the same time use the resources and competencies developed while working together. We test in an experimental study whether working in MTM as compared to a single team yields more individual performance benefits in estimation tasks. Our results fully support the group-to-individual (G-I) transfer of learning, yet the hypothesized benefits of knowledge variety and broader access to meta-knowledge relevant to the task in MTM as compared to single teams were not supported. In addition, we show that individual estimates improve only when members are part of groups with low or average collective estimation errors, while confidence in individual estimates significantly increases only when the collective confidence in the group estimates is average or high. The study opens valuable venues for using the dynamic model of G-I transfer of learning to explore individual learning in MTM.

## Introduction

Multiple team membership (MTM) was defined as the simultaneous allocation of individuals (i.e., employees, students, etc.) across different teams and tasks (O'Leary et al., 2011) or as a form of membership interdependence across different teams (Margolis, 2020). For individual members, being part of one team or being part of multiple teams generates a social learning context through the unfolding interpersonal interactions. Given the diversity of team contexts, the individual members are exposed to in MTM, such a context is expected to offer more learning and development opportunities than single team membership (O'Leary et al., 2011; Chen et al., 2021). While team literature has focused extensively on studying (single) team membership and the role it plays for the development of individual decision competencies (Curseu et al., 2015), relatively little is known of the effects of MTM on such phenomena (O'Leary et al., 2011). This is a gap in the literature that requires further exploration especially given that MTM is frequently used in organizations (Chen et al., 2019; Berger et al., 2021) and higher education as a form of work organization that allows the flexible use of human resources across a variety of projects and teams. Moreover, MTM was often advertised as a development (i.e., learning) opportunity for individuals. In this paper, one of our aims is to look at the effects of single vs. multiple team membership on individual performance gains in estimation tasks.

MTM was predicated to have both positive and negative effects on individual performance and well-being (O'Leary et al., 2011; Pluut et al., 2014; Rapp and Mathieu, 2019; Berger et al., 2021; Chen et al., 2021) and during the last decade, a growing number of empirical studies have examined the benefits and detriments of MTM (Margolis, 2020). The role enrichment associated with simultaneously working in different team contexts is among the most notable individual benefits of MTM. Interacting with diverse others is expected to foster individual learning and yield important cognitive benefits for individuals. Literature to date has explored such beneficial effects in field studies (Chan et al., 2020) yet little attention was shown to directly testing the cognitive gain associated with MTM in tasks with objective performance indicators. Furthermore, the field studies were not able to directly compare the effects of single vs. multiple team membership on individual performance gains. We find this comparison important (especially in an experimental design) in order to understand the real impact of team membership on individual performance gains. Hence, our study aims to address this gap in the literature and attempts to answer the following research question: Does MTM improve individual performance in estimation tasks, beyond the mere effect of single group membership? It is to be noted that we focus on individual performance and the extent to which it improves after individuals are exposed to one of the two conditions: single vs. multiple-team membership. We acknowledge as well that improvement in individual performance after group interaction can also be conceptualized as learning while following the group to individual transfer of learning approach (Gustafson et al., 1973; Brodbeck and Greitemeyer, 2000; Schultze et al., 2012).

We report here an experimental study in which we evaluated individual performance and confidence in the answers provided in an estimation task prior and after group estimations and we manipulated team membership such that some participants were allocated to the same team for all estimation tasks (single team condition), while other members were allocated to a different team separately for each estimation task (multiple team membership condition). Such a design allowed us to control the type of task across different team contexts and to estimate the cognitive individual gain in function of the type of membership, namely single team membership vs. multiple team membership.

We contribute to existing research in several ways. First, we extend the group learning model introduced by Brodbeck and Greitemeyer (2000) by adding the context of multiple team membership as a social setting that can shape the effectiveness of group to individual and individual to group transfer of learning. Second, we contribute to the literature on MTM by controlling the type of task performed by the participants and as such being able to compare the effectiveness of learning in MTM as compared to single group settings. Third, our study has important practical implications pointing to the relevance of fostering group synergy as a key factor for stimulating performance and confidence gains of individual members in single groups as well as in the MTM context.

## Theoretical Background and Hypotheses

Studies looking at single-team contexts have repeatedly documented the performance benefits that individuals have as a consequence of being part of a team. For example, literature on group synergy shows that groups outperform the average performance of their individual members in problem solving and judgment tasks due to a greater information pooling and sharing, greater error correction capabilities, and the development of meta-knowledge related to the task (Larson, 2010; Hinsz, 2015). Individual group members benefit from this elevated information processing capacity of teams by refining and improving their own individual performance in cognitive tasks building on the emerging insights from the similar tasks previously performed with the group. In the dynamic model of group performance (Brodbeck and Greitemeyer, 2000), such an elevated individual performance following group discussion has been labeled as group-to-individual (G-I) transfer of learning. While discussing with others in groups, individual group members may experience (internal) cognitive conflicts by being exposed to different and sometimes divergent points of view. The resolution of such cognitive conflicts increases the depth of information processing and ultimately the accuracy of individual judgments (Nemeth and Kwan, 1987; Brodbeck and Greitemeyer, 2000). Next to the task-related benefits associated with group discussions, individual group members may develop meta-knowledge related to how to approach the task and to successfully engage in knowledge sharing and evaluation individually and in groups (Hinsz, 2015). Such meta-knowledge may be acquired via explicit or implicit processes. For instance, McMahon and Ford (2013), showed that team leaders may engage in explicit articulations of “rules of thumb” that they use for pattern recognition, discovery, and problem solving, and that communicating about such heuristics further enhances team members' creative performance. McMahon and Ford (2013) labeled the process as leader heuristic transfer (LHT). Similar processes of heuristic transfer whereby individuals share their experience-based “tips and tricks” for approaching a variety of tasks can occur at the team level irrespective of the members' status.

Literature to date provides extensive empirical support for the knowledge-related (e.g., members compare their judgments with the ones expressed by others) and meta-knowledge related (e.g., members learn how the best performing group members make their judgments or how the group refines these individual judgments) benefits of group discussions or the G-I transfer of learning (Gustafson et al., 1973; Brodbeck and Greitemeyer, 2000; Schultze et al., 2012; Lippold et al., 2021). However, all of these studies are conducted while looking at membership in one team only or they compare the G-I transfer of learning in interacting vs. nominal groups. Little to no empirical evidence exists in support of G-I transfer of learning in multiple teams. In principle, by allowing access to a richer pool of knowledge and expertise, MTM should enhance both knowledge-related and meta-knowledge related benefits of group interactions.

In their conceptual analysis, O'Leary et al. (2011) argued for a non-linear association between the number of teams an individual is a part of and individual learning. The number of teams is expected to have benefits (increased exposure to a variety of viewpoints, opinions, and knowledge) as well as detriments (decreased depth of information processing, reduced attention span to informational contents, and decreased encoding time) for individual learning. Little empirical evidence for this non-linear association has emerged so far with two notable exceptions that have documented an inverted U shape association between the number of MTM and individual performance (Chan, 2014; Bertolotti et al., 2015). More recent empirical evidence has showed that moderate cognitive diversity in teams has beneficial effects on team learning (Aggarwal et al., 2019) and that the membership variety in MTM is positively associated with individual learning (Chan et al., 2020). Such a fostering effect for individual learning is likely to be a reflection of the G-I transfer of learning. Therefore, we build on the G-I transfer of learning (Brodbeck and Greitemeyer, 2000) to argue that individual members use the other group members as information sources and during the group discussion they develop more accurate insights into the estimation tasks and, as a consequence, they improve their individual performance (Stern et al., 2017). In line with this model, if individuals are exposed to more groups, they have access to broader cognitive resources, to more meta-cognitive strategies for dealing with the task, and therefore the G-I transfer of learning is fostered in MTM contexts. Given these arguments stemming from the G-I transfer of learning and the MTM literature we hypothesize the following:

Hypothesis 1: Individual performance in estimation tasks increases after group interaction.
Hypothesis 2: The performance gain will be higher for individuals who are part of MTM in comparison to individuals who are part of single team membership.

Studies that contrasted individual and group decision tasks show that in general groups tend to report higher levels of confidence in their choices or judgments compared to their individual members working alone (Sniezek, 1992). Therefore, beyond objective performance, confidence or the evaluation of the cognitive products that individuals and groups generate (i.e., an estimation of the probability of a certain event, a decision, etc.) is also important as they frequently operate in conditions of uncertainty. In such situations, team members cannot identify right away whether their answer is correct, by evaluating it against objective criteria. Hence, it is the level of confidence (i.e., beliefs in their accuracy) they experience with respect to the estimation provided or the decision made that is likely to influence whether they further act upon it or not.

Next to the cognitive influence of groups on individuals, groups (including multiple teams) are also sources of social identification (Rapp and Mathieu, 2019) and often group members use each other to validate their views (informational influence) and gain confidence in their own opinions (Deutsch and Gerard, 1955). Previous studies have shown that confidence in estimates in a quantitative task changes when group members are exposed to different opinions of the other group members (Rowe et al., 2005). In groups, individuals have the opportunity to contrast and compare their knowledge and opinions with the ones belonging to the other group members and adjust them based on these comparisons. We, therefore, expect that, given the same estimation task, the confidence in the individual judgments made after the group interaction substantially increases.

Hypothesis 3: Individual confidence in the estimation tasks increases after group interaction.

In a recent empirical study on MTM, Chen et al. (2019) show that leadership influences experienced in a particular team context are carried over to other team contexts. Moreover, the results reported in Rapp and Mathieu (2019) show no significant decrease in identification with the teams as the number of MTM increases, illustrating that members tend to identify rather equally with all the different teams they work in. It is therefore not unreasonable to assume that the social support for one's views is carried over and amplified in MTM contexts. On the other hand, in a recent empirical study using estimation tasks, individuals improved their performance after a single exposure to the group (Stern et al., 2017) and, in line with this evidence, we would expect that MTM is actually taxing on the confidence of the individuals in their own judgments. We, therefore, formulate the last hypothesis concerning the impact of MTM on changes in confidence as a set of competing claims.

Hypothesis 4a: The increase in confidence is stronger for individuals who are part of MTM than for individuals who are part of a single team.
Hypothesis 4b: The increase in confidence is weaker for individuals who are part of MTM than for individuals who are part of a single team.

## Methods

### Sample and Procedure

Our sample consisted of 115 students (85 women and 30 men, with an average age of 20.71 years old) enrolled in social psychology courses at a large European university. They participated in the study in exchange for course credits. The procedure of the study consisted of three major steps.

In the first step, students were asked to give their individual answers to four estimation tasks: (i) percentage of women in the Senate, (ii) percentage of women graduate in the field of science and technology, (iii) percentage of male in the board of major national companies listed at the stock exchange, and (iv) percentage of national companies that have at least a woman in top management. The correct estimates were obtained from The Global Gender Gap Report of the World Economic Forum for 2021. They had in total 10 min to complete the task and a brief survey individually. We used the absolute difference between the individual estimate and the correct estimate as a performance indicator (such an absolute difference reflects the absolute estimation error across the four tasks). At the end of the task, they had to state their confidence in their estimate. We build on the insights from Sniezek (1992) stating that next to estimation accuracy, the confidence in the estimations is also a relevant metric and, in line with this stream of research, we used the following item to measure confidence “On a scale from 0 to 100, to what extent are you confident that you gave the correct answer in the previous estimation task?”

In the second step, all participants were allocated to one of the two experimental conditions. In the single group condition, they were asked to join a group consisting of the same members that had the task to discuss and make estimates through agreement on all four tasks (same as in step 1), make a group estimate for each task, and then evaluate as a group (through agreement) their collective confidence in each of these estimates. Groups had in total 10 min per round (40 min in total) in order to complete the estimation tasks.

The number of simultaneous teams in the MTM has important implications for individual outcomes. O'Leary et al. (2011) theorized an inverted U shape association between the number of teams and individual learning, yet the empirical evidence to date did not identify an unequivocal inflection point (Margolis, 2020). Empirical studies report an inflection point that can vary from 2 to 4 teams (Fricke and Shenbar, 2000; Chan, 2014) and up to 9 teams (Bertolotti et al., 2015). For this study, we decided to use four simultaneous teams, in order to capture the maximum cognitive benefits likely to emerge while simultaneously working in multiple teams.

As such, in the MTM condition, each participant was a member of four different groups. Members changed the group for each of the four estimation tasks (e.g., in the first group, they solved only the first estimation task; in the second group, they solved only the second estimation task and so on). Each group had a size of three members. For each estimation task and the rating of the confidence in their estimation, groups spent a total of 10 min. The group estimation error was computed as the average estimation errors across the four estimation tasks performed together with a group, either in the same team (single group condition) or in different teams (MTM condition).

In the third step, after the single group/MTM sessions, participants were asked to read again the four estimation tasks that they were also exposed to in steps 1 and 2 and to give again an individual answer to the tasks. They had in total 10 min to complete this step. At the end of the task, they had to state their confidence in their estimate. We used the same item mentioned above to measure confidence.

**Figure 1:**
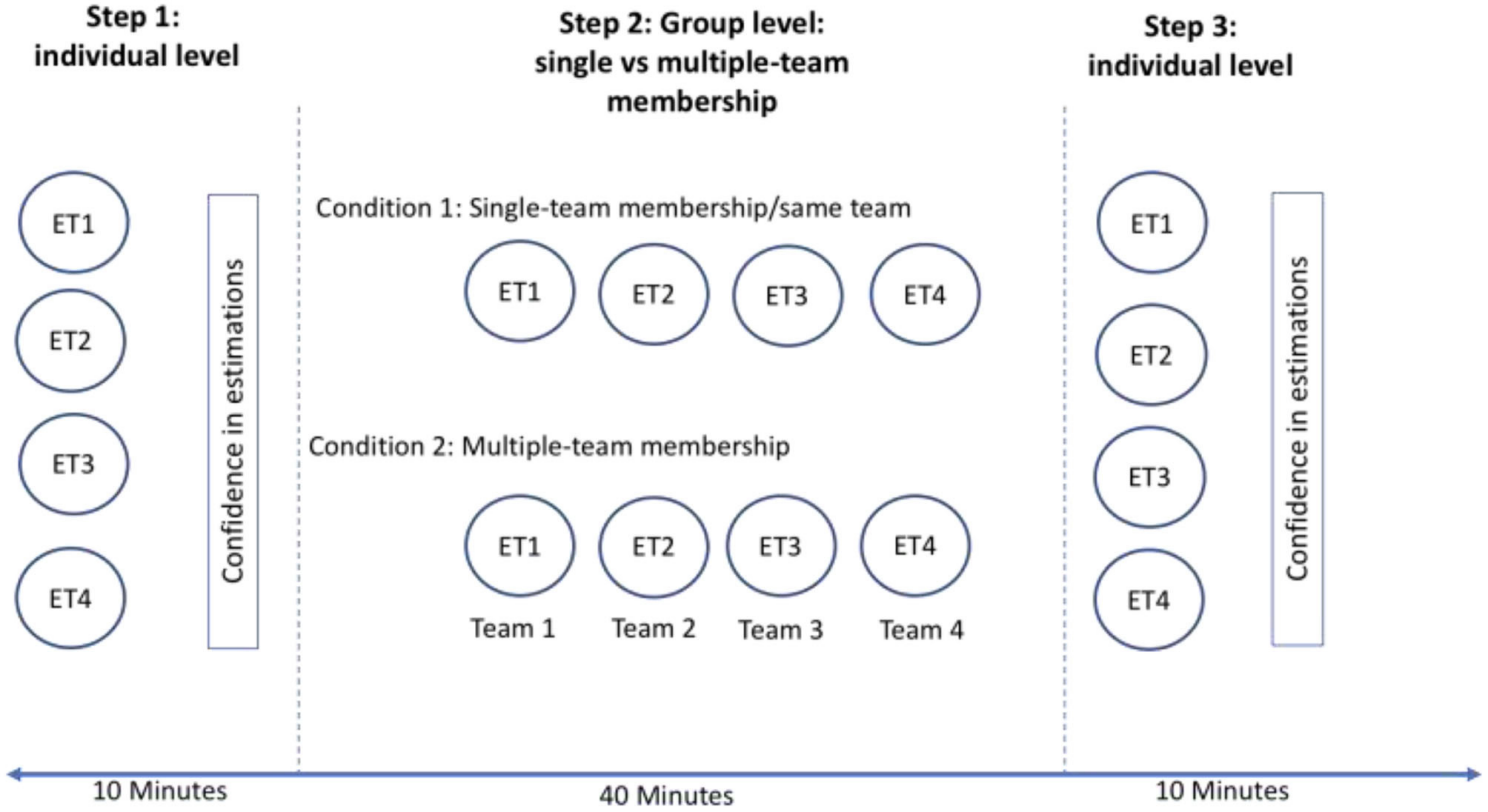
Experimental design. ET1, estimation task 1; ET2, estimation task 2; ET3, estimation task 3; ET4, estimation task 4; in the single team membership condition participants performed all estimation tasks in the same team; in the multiple team membership condition participants had a different team for each of the four estimation tasks.

The confidence index was computed by averaging the confidence estimates across the four different tasks, while learning was estimated as a decrease in the estimation errors from the initial (step 1) to the post-group session (step 3). The design of the experiment can be visualized in Figure 1.

At the end of the task, we asked participants to fill in a short survey containing demographic variables (gender, age) and the 18-item version of the Need for Cognition (NFC) scale (Cacioppo et al., 1984) adapted and translated for the national population by Curşeu (2004). Cronbach's alpha for the NFC scale is 0.74 and the omega index based on the items loading derived from a confirmatory factor analysis (Hayes and Coutts, 2020) is 0.78 indicating a sufficient internal reliability of the scale. NFC was included as a control variable because the need for cognition is positively correlated with cognitive task performance (Cacioppo et al., 1996) as well as with advice seeking from teammates during group interactions (Curşeu, 2011). Also, the need for cognition was found to moderate the relation between multiple project team membership variety and individual learning (Chan et al., 2020).

## Results

Table 1 presents the means, standard deviations, and correlations among the variables used in this study.

**Table 1:** Means, standard deviations, and correlations.

	**Mean**	**SD**	**1**	**2**	**3**	**4**	**5**	**6**	**7**	**8**	**9**
1. Gender	0.7391	0.44103	1								
2. Experimental condition	0.6121	0.48939	−0.111	1							
3. Age	20.7130	2.31988	−0.100	0.001	1						
4. Need for cognition	3.5945	0.43057	0.047	−0.063	−0.145	1					
5. Average individual EE pre	16.6035	6.22288	0.002	−0.103	−0.106	−0.170	1				
6. Average individual EE post	12.9280	5.87425	−0.139	−0.163	0.010	−0.173	0.440^**^	1			
7. Individual confidence estimation pre	54.9737	18.24190	−0.210^*^	0.071	0.063	−0.059	−0.174	−0.051	1		
8. Individual confidence estimation post	65.8231	13.43485	−0.086	0.075	0.122	−0.050	−0.128	−0.271^**^	0.381^**^	1	
9. Average group EE	11.9339	5.55751	−0.113	−0.113	−0.010	−0.164	0.420^**^	0.820^**^	−0.063	−0.223^*^	1
10. Average group confidence	66.2782	12.61246	−0.035	0.028	−0.028	−0.003	−0.035	−0.237^*^	0.204^*^	0.782^**^	−0.226^*^

*N = 115; EE, estimation error; pre, before group interaction; post, after group interactions; gender is coded as a dummy variable with 0 = male, 1 = female; experimental condition is a dummy variable with 0 = single group membership, 1 = MTM membership.*

* *p < 0.05,*

** *p < 0.01*.

**Figure 2:**
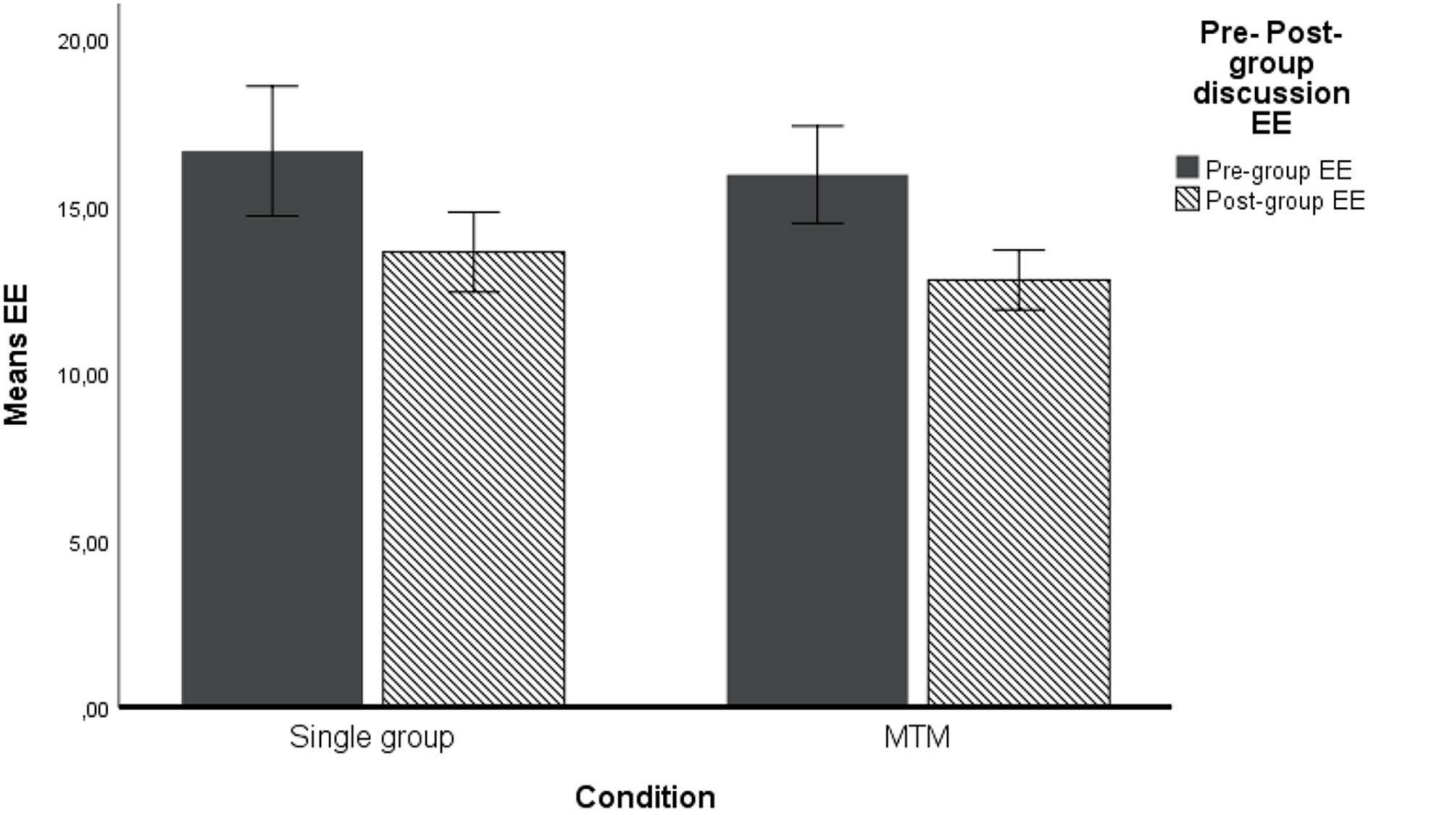
Means estimation error (EE) pre- and post-group discussion. Covariates appearing in the model are evaluated at the following values: Age = 20.7925. NFC = 3.5736. AVGErrGR = 12.0316. Error bars: 95% CI.

We used ANOVA for repeated measures to test the study hypotheses. This analytical procedure is appropriated given the nature of our design that includes both within as well as between participant components. As a within-subjects factor, we used the estimation errors before the group interactions and after the group interactions, while as a between factor we used the experimental condition (MTM vs. single group membership). Moreover, as covariates we used gender, age, NFC, and the average group estimation errors in the four estimation tasks. We computed the average group estimation error as the average estimation error of the nominal group (or groups in MTM condition, where each participant was part of four different groups) an individual was part of in the second step. First, we ran the analyses without the control variables and the results show a significant decrease in the estimation errors, with *F*_(1, 104)_ = 27.31 (*p* < 0.0001) η^2^ = 0.21, observed power is 0.99, therefore the first hypothesis was supported. However, the interaction between the group membership type and the within subjects factor (MTM vs. single group membership) was not statistically significant, with *F*_(1, 104)_ = 0.26 (*p* = 0.61) η^2^ = 0.002, observed power was 0.08, therefore Hypothesis 2 stating that the performance gain will be higher for individuals part of MTM was not supported. The results are available in Table 2 and are depicted in Figure 2.

**Table 2:** Means and standard deviations (SD) for estimation errors and confidence.

**Dependent variable**	**Experimental condition**	**Pre-group mean**	**Pre-group SD**	**Post-group mean**	**Post-group SD**
Estimation error	Single group	17.2167	6.25794	14.1846	5.90027
	MTM	15.9381	6.04560	12.2530	5.81069
Confidence	Single group	54.2188	17.44884	64.5250	13.98543
	MTM	56.8308	17.30551	66.9472	13.18626

We ran the analyses with the covariates and the pattern of results remains the same. None of the control variables (including NFC) had a significant association with the pre- or post-group estimation errors. The difference between the pre-group and post-group estimation errors was significant, with *F*_(1, 100)_ = 5.33 (*p* = 0.02) η^2^ = 0.05, observed power was 0.63, while the interaction of the within and between subjects factor was not significant, with *F*_(1, 100)_ = 0.01 (*p* = 0.92) η^2^ = 0.000, observed power was 0.05. From the covariates, only the average group estimation error had a significant effect on the cognitive gain *F*_(1, 100)_ = 103.15 (*p* < 0.0001) η^2^ = 0.51, observed power was 1. Moreover, the interaction between the pre-post group discussion estimation errors and the average group estimation error was significant, with *F*_(1, 100)_ = 13.58 (*p* < 0.0001) η^2^ = 0.12 observed power was 0.95. In order to further explore this interaction effect, we used the MEMORE procedure described by Montoya (2019) that allows the estimation of moderation effects for repeated measures. Similar to the ANOVA analyses, the effect of the experimental condition is not significant. The effect of average group estimation error on the difference between the pre- and post-group individual estimation was negative and significant *B* = −0.40, *SE* = 0.11 *p* = 0.003, [−0.61; −0.19] showing that the cognitive gain for individuals in groups that make high estimation errors is lower than for individuals in groups that make low estimation errors. The association between average group estimation errors and individual estimation errors in the pre-group stage was positive and significant *B* = 0.46, *SE* = 0.10, *p* < 0.0001 [0.26; 0.65], showing that groups composed of individuals with low estimation errors also tend to make low estimation errors as a group. In the post-group stage, the effect of the average group estimation errors on individual estimation errors was also positive and significant *B* = 0.85, *SE* = 0.06 *p* < 0.0001 [0.74; 0.97], supporting a significant group-to-individual transfer of learning. The conditional effects presented in Table 3 show that the cognitive gain is significant only when the average group estimation errors are low or average. When groups make significantly more estimation errors, group members do not benefit from the group, in other words under these conditions, the group-to-individual transfer of learning is not significant.

**Table 3:** Conditional effects.

**Average group EE**	**Experimental condition**	**Pre- post group EE**		**Pre- post group confidence**	
		**B (SE)**	**CI**	**B (SE)**	**CI**
Low	Single group	5.59^***^ (1.19)	[3.25; 7.98]	−3.78 (2.98)	[–9.68; 2.12]
Low	MTM	5.70^***^ (0.91)	[3.89; 7.50]	–3.08 (2.59)	[−8.21; 2.06]
Average	Single group	3,38^**^ (0.97)	[1.46; 5.30]	−10.62^**^ (2.56)	[−15.71; −5.54]
Average	MTM	3.48^***^ (0.73)	[2.02; 4.95]	−9.92^***^ (2.01)	[−13.91; −5.93]
High	Single group	1.16 (1.08)	[−0.99; 3.31]	−17.47^***^ (3.05)	[−23.52; −11.41]
High	MTM	1.27 (0.98)	[−0.67; 3.20]	−16.76^***^ (2.53)	[−21.78; −11.73]

*Unstandardized regression coefficients are shown with standard errors between parentheses;*

*** *p < 0.001,*

** *p < 0.01. EE, estimation error*.

**Figure 3:**
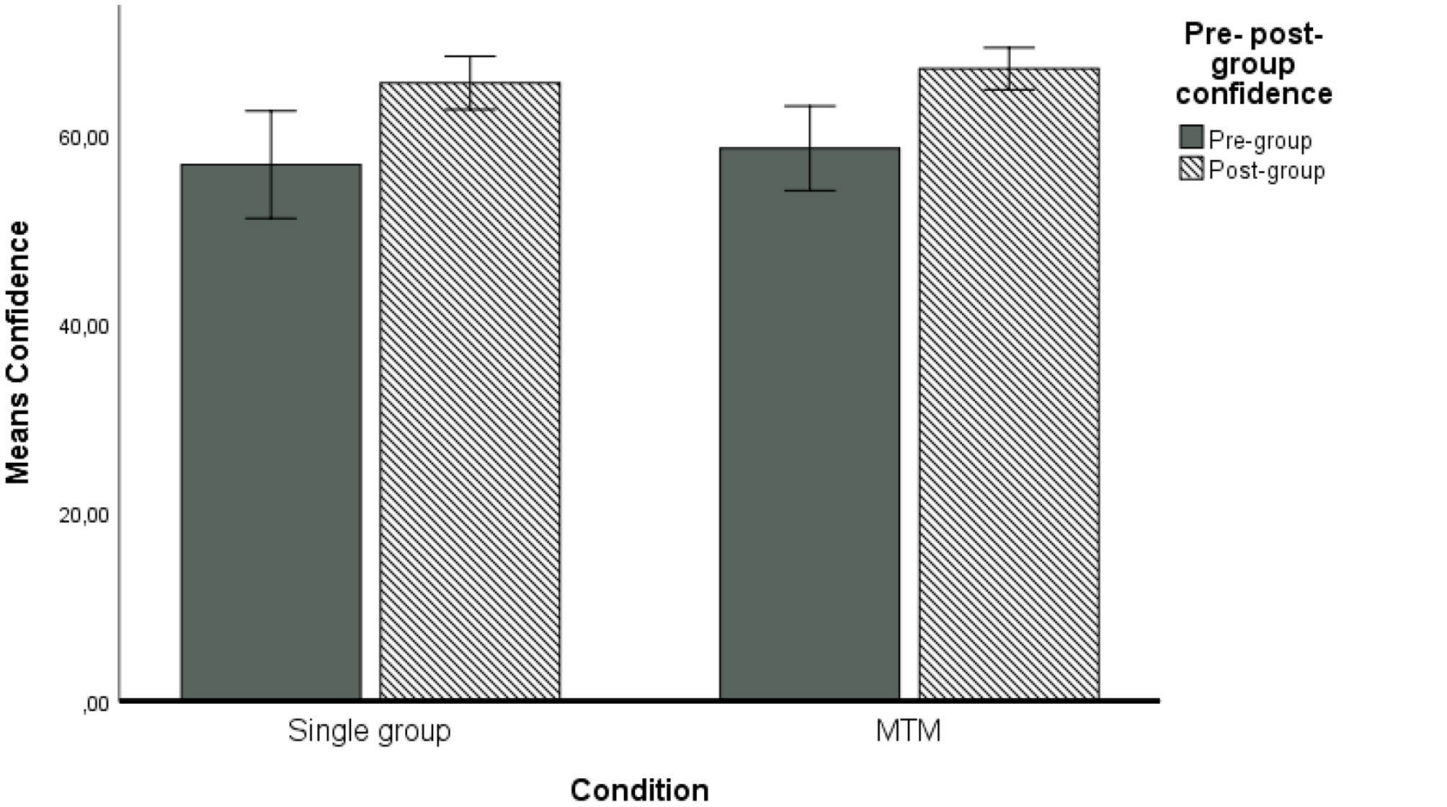
Means confidence in estimates pre- and post-group discussion. Covariates appearing in the model are evaluated at the following values: Age = 20.8095. AVGConGR = 66.5346. NFC = 3.5760. Error bars: 95% CI.

We used a similar procedure to test the third and the fourth hypotheses and we started with a repeated measures ANOVA analysis without any covariates. The results show that the change in confidence from pre- to post-group estimation was significant, with *F*_(1, 103)_ = 33.66 (*p* < 0.0001) η^2^ = 0.25, observed power was 1.00, therefore supporting the third hypothesis. The interaction of the within subjects (pre- vs. post-group estimation) and the between subjects (the experimental condition with single group vs. MTM) was not significant, with *F*_(1, 103)_ = 0.003 (*p* = 0.96), η^2^ = 0.003, observed power was 0.05, showing that neither MTM (Hypothesis 4a), nor the single group membership has the expected benefit for the confidence in the estimates (Hypothesis 4b). The results are presented in Figure 3 and did not yield support for Hypothesis 4.

**Figure 4:**
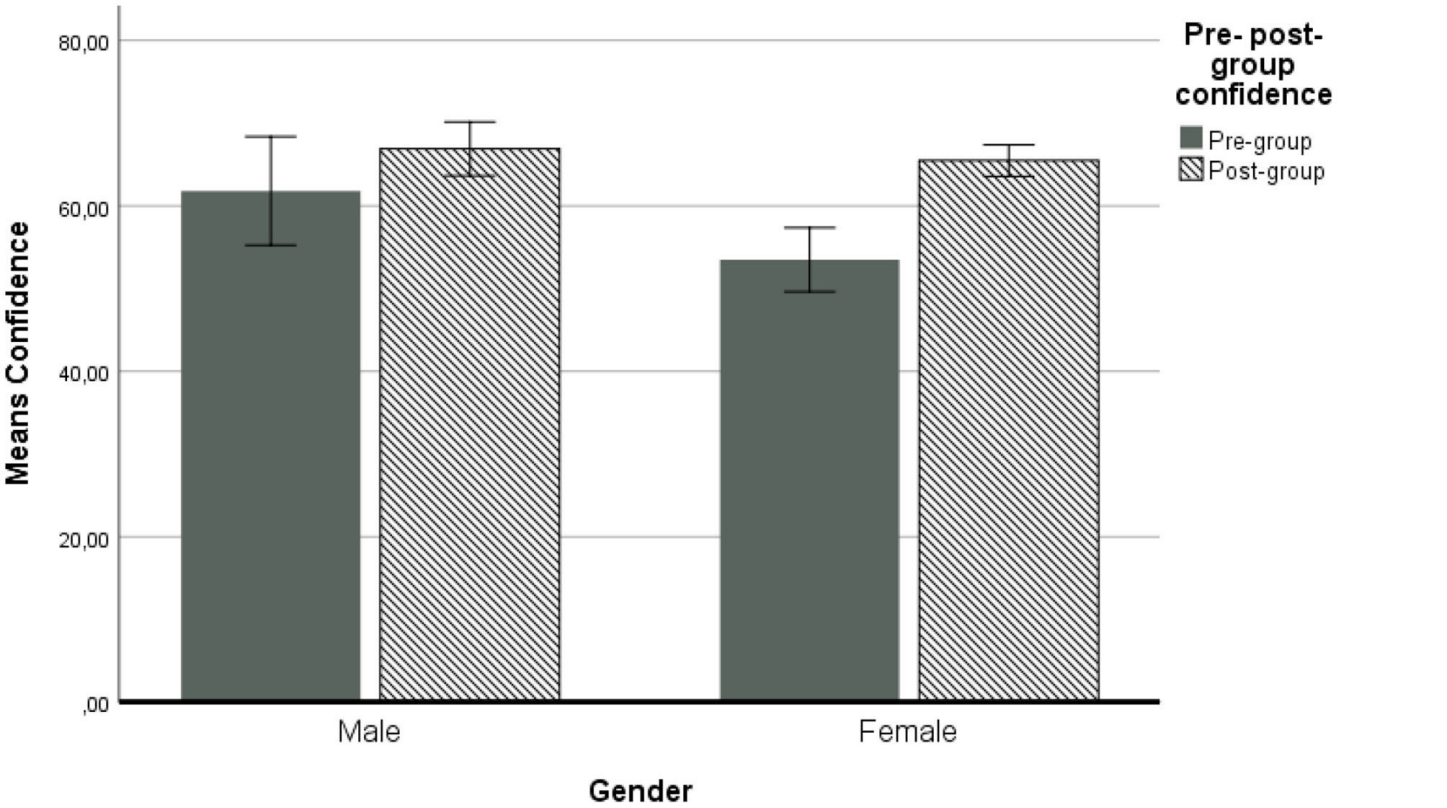
Gender differences in confidence for pre- and post-group discussion. Covariates appearing in the model are evaluated at the following values: Age = 20.8095. AVGConGR = 66.5346. NFC = 3.5760. Error bars: 95% CI.

The repeated measures ANOVA with covariates shows similar results. From the control variables, age and NFC had no significant association with the confidence in the estimates. Gender however, had a significant main effect *F*_(1, 99)_ = 4.15 (*p* = 0.04) η^2^ = 0.04, observed power was 0.52 showing that, in general, men (*M* = 65.05) tend to be more confident in their estimates than women are (*M* = 59.15). This comes in line with previous results showing that the percentage of women within groups is associated with group underestimation of performance (Meslec and Aggarwal, 2018). Gender also had a marginally significant interaction with the within subjects factor *F*_(1, 99)_ = 3.64 (*p* = 0.05) η^2^ = 0.04, observed power was 0.47 and this interaction effect is presented in Figure 4.

Moreover, the average group confidence in the group level estimates had a significant positive effect on the individual confidence with *F*_(1, 99)_ = 45.25 (*p* < 0.0001) η^2^ = 0.31, observed power was 1. The interaction effect of the within subjects factor (pre- vs. post-group confidence) with the average group level confidence was also significant with *F*_(1, 99)_ = 19.79 (*p* < 0.0001) η^2^ =0.17, observed power is 0.99. In order to further explore this interaction effect, we used the MEMORE procedure (Montoya, 2019) as in the previous set of analyses. The association between group confidence in collective choice and the post- to pre-group difference in individual confidence was positive and significant *B* = 0.54, *SE* = 0.13, *p* < 0.0001 [0.29; 0.79] showing that the increase in individual confidence from pre- to post-group discussions was directly proportional with the group confidence. To conclude, the conditional effects presented in Table 3 show that the average group confidence significantly impacts on the increase in individual confidence only at high and average levels.

Table 4 includes an overview of the study hypotheses and whether they received empirical support.

**Table 4:** Overview of the hypotheses tested in the study.

**Study hypotheses**	**Status**
Hypothesis 1: Individual performance in estimation tasks increases after group interaction.	Supported
Hypothesis 2: The performance gain will be higher for individuals part of MTM in comparison to individuals part of single team membership.	Not supported
Hypothesis 3: Individual confidence in the estimation tasks increases after group interaction.	Supported
Hypothesis 4a: The increase in confidence is stronger for individuals part of MTM than for individuals part of single team.	Not supported
Hypothesis 4b: The increase in confidence is weaker for individuals part of MTM than for individuals part of single team.	Not supported

## Discussion

Our study aimed to explore the extent to which being part of MTM has cognitive benefits in estimation tasks beyond the cognitive benefits associated with belonging to a single team. Our results for Hypothesis 1 replicate general findings in the G-I transfer of learning and show that the quality of individual estimates increases after group members discuss the tasks in a single group or in multiple groups. This may be due to having acquired better strategies to tackle the task following group interactions [i.e., meta-knowledge (Hinsz, 2015)] and or due to the exposure to multiple information sources (Stern et al., 2017) and acquiring more metric (i.e., knowledge about the scale a target is measured on) and mapping knowledge (i.e., knowledge about the position of specific targets on a scale) (Lippold et al., 2021). By engaging in task conversations with members of the groups who possibly hold different perspectives on the task at hand, individual group members are likely to experience (internal) cognitive conflicts. Solving such cognitive conflicts requires in-depth information processing which, in turn, increases the accuracy of individual judgments in the estimation tasks following group discussions.

Our results for Hypothesis 2, however, did not support the superiority of the MTM context for individual learning in estimation tasks. We used a single type of task across the simultaneous teams, therefore, when task variety is controlled, the hypothesized benefits of exposure to a broader knowledge repertoire in MTM do not yield learning benefits beyond the mere participation in a single group. Individual differences such as the need for cognition (i.e., individuals' tendency to engage in and enjoy thinking) (Chan et al., 2020), could partially account for this finding such that individuals high in need for cognition might benefit more from working in a MTM setting. Having established this baseline for G-I transfer of individual learning in MTM vs. single groups, we believe it is important to further explore in experimental designs the way in which the variety of tasks performed in different teams could impact on individual learning. Because G-I transfer of learning in single team contexts was well-established in a variety of cognitive tasks (Brodbeck and Greitemeyer, 2000; Lippold et al., 2021), future studies could use different types of tasks (e.g., estimation, problem-solving, decision-making, and creativity) and check the extent to which such variety is likely to bring benefits when participants perform these different tasks in different groups as opposed to the same group. Another interesting avenue for future research is the adaptation of the “hidden-profiles” type of tasks to the MTM context. Research on the “hidden-profile” has pointed to the tendencies of team members to focus on shared rather than unique (distributed) information during group debates (Lu et al., 2012), thus reducing the variety of the information pool that eventually supports individual learning. Expanding the “hidden-profile” paradigm to MTM contexts would, on the one hand, answer the call for a more dynamic perspective to this paradigm (Sohrab et al., 2015) and, on the other hand, it would allow the more systematic exploration of meta-cognitive transfer across multiple teams using established paradigms of group research (Margolis, 2020). Finally, in our study, we look mainly at the extent to which individuals improve their performance as a result of being part of one or multiple teams. Teams, however, can experience different dynamics in their interaction. They may have to deal with relational conflict, information hiding or an excessive focus on maintaining a positive atmosphere and cohesiveness. Or, they could experience adequate levels of psychological safety, focus on solving disagreements, and addressing the task at hand. Extant research (Lee et al., 2018; Wiese and Burke, 2019; Kim et al., 2020) is generous in highlighting the differential impact of such process losses vs. gains on team outcomes (i.e., learning, performance, viability, commitment, etc.). Further studies could take a look at the effects that these interaction patterns have on the group to the individual transfer of learning.

In the additional moderation analyses using MEMORE, we also illustrated the individual-to-group (I-G) transfer of learning as our results show clearly that group estimation errors are positively associated with individual estimation errors in the pre-group stage. This result is in line with the arguments of the Brodbeck and Greitemeyer (2000) group learning model that integrates the two forms of transfer, namely, I-G and G-I, to explain how social learning unfolds in groups. Although this type of transfer was not the main aim of our study and it was not directly hypothesized, we believe it is important to mention it as one of the key elements that contributes to the MTM literature. Because one's education, expertise, and organizational rank are important predictors for the number of MTM (Margolis, 2020), future research could explore the interdependencies between individual cognitive competencies (problem-solving or decision-making competencies) and the number of MTM in relation to learning, performance or other individual level outcomes.

Our results also show, in line with Hypothesis 3, that individuals improve their levels of confidence regarding how well they solved the task as a consequence of their group experience. This result indicates that, through social comparison processes, individuals manage to improve not only their knowledge in relation to the task but also their meta-knowledge related to whether they think they did well or not in the task. Finally, we did not find empirical support for Hypothesis 4. Data shows that confidence does not improve when individuals are members of multiple teams as compared to being a member of a single team.

An emerging result concerns the role of group estimation errors and average confidence in group judgments showing that cognitive gains occur only when the groups have a low or average level of estimation errors, while the boost in confidence occurs only when the groups express average or high levels of confidence. These results add to the G-I transfer of learning and to the MTM literature showing that group synergy is one of the key antecedents for the expected cognitive benefits of MTM. Previous research showed that members of groups that achieve synergetic performance (the collective performance exceeds the performance of its average and best members) have superior cognitive gains as compared to members of non-synergic groups (Curseu et al., 2015). Future research could therefore explore the extent to which group performance level and differences across teams impact one's individual learning in MTM. Moreover, we call for more research on the mechanisms that explain the emergence of cognitive synergy in groups. The identification of such mechanisms has important applications for improving group performance as well as the effectiveness of social learning in groups and MTM settings.

## Limitations

Our study has some relevant contributions to the MTM literature and opens new venues for the exploration of the learning outcomes stemming from MTM, yet it also has some limitations. First, due to the convenience for running an experimental study, we have used a standard estimation task and a sample of students. Previous research showed that results may differ if the study sample includes students or non-students (Gordon et al., 1986; Peterson, 2001; Peterson and Merunka, 2014). However, using student samples is adequate when testing whether a phenomenon can occur as long as the sample is not biased with respect to the research question (Calder et al., 1981; Shen et al., 2011). The similarity of the (student) sample with the target population is not required for highlighting a particular relation between the study variables under these circumstances. This is exactly the case of this study, as the aim was to test whether MTM can produce a higher G-I transfer of learning as compared to being part of a single team, while controlling for NFC. However, in order to ensure generalizability, our results need to be replicated in other settings, on different samples and with other types of tasks.

Second, although we have controlled for cognitive motivation (NFC is associated with performance in cognitive tasks as well as with advice seeking in groups), other cognitive competencies could have influenced the performance in the estimation tasks used in this study. Third, our study used the same procedure for the single group and MTM conditions (participants made all four estimates individually and then collectively in the two separate conditions), yet alternative approaches as the ones described in Schultze et al. (2012) could be used to fully discern between I-G and G-I transfer of learning. Future studies could further build on the literature on the G-I transfer of learning and adapt alternative designs to disentangle the G-I and I-G transfer in MTM. Finally, estimation tasks are particular types of decision-making tasks and the results cannot be generalized to the overall decision-making competencies, therefore future studies could use more comprehensive tasks that capture global decision-making competencies and explore the G-I and I-G transfer of such competencies associated with MTM.

## Conclusions

Our study builds on research on MTM and G-I transfer of learning to test in an experimental design the MTM advantage over single group membership in improving individual performance in estimation tasks. Although our results did not support the hypothesized benefits of MTM, we make some relevant contributions to the literature on MTM. First, we show that when the type of task is controlled, the expected learning and performance benefits of MTM do not occur. Second, our research shows that the accuracy of pre-group individual estimates is positively correlated with the accuracy of group estimates pointing to significant effects of I-G transfer of learning, a process that was rather overlooked in MTM research so far. Third, we show that individuals benefit from group discussions only when groups have average to high performance in estimation tasks, pointing toward the key role of G-I transfer of learning. To conclude, our study shows that the G-I and I-G transfer of learning stipulated in dynamic group learning research (Brodbeck and Greitemeyer, 2000; Schultze et al., 2012) should be explored jointly in order to disentangle the benefits of MTM for individual learning and other performance-related outcomes.

## Data Availability Statement

The raw data supporting the conclusions of this article will be made available by the authors, without undue reservation.

## Ethics Statement

The studies involving human participants were reviewed and approved by Institutional Review Board, Babeş-Bolyai University. The patients/participants provided their written informed consent to participate in this study.

## Author Contributions

OF, PC, and NM were involved in the study design, data collection and analysis, writing, and editing the manuscript. All authors contributed to the article and approved the submitted version.

## Conflict of Interest

The authors declare that the research was conducted in the absence of any commercial or financial relationships that could be construed as a potential conflict of interest.
